# Supplementary material for: Brain network dynamics in high-functioning individuals with autism
Source: Nat Commun. 2017 Jul 5;8:16048. doi: 10.1038/ncomms16048 (PMC5504272; doi:10.1038/ncomms16048)
Supplement: Supplementary Information [file ncomms16048-s1.pdf]

File Name: Supplementary Information
Description: Supplementary Figures, Supplementary Tables and Supplementary References

File Name: Peer Review File
Description:

Supplementary Figure 1

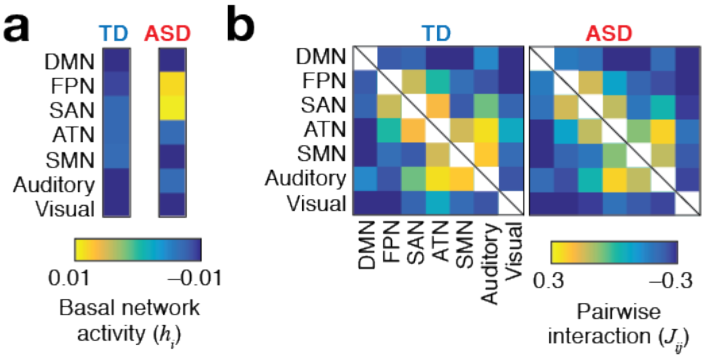

Results of fitting of a pairwise maximum entropy model.

As results of accurate model fitting, we obtained two indices representing hypothetical basal network activity (**a**) and pairwise interaction (**b**). Using these indices, we then calculated energy values of all the possible brain activity patterns, and built energy landscapes.

Supplementary Figure 2

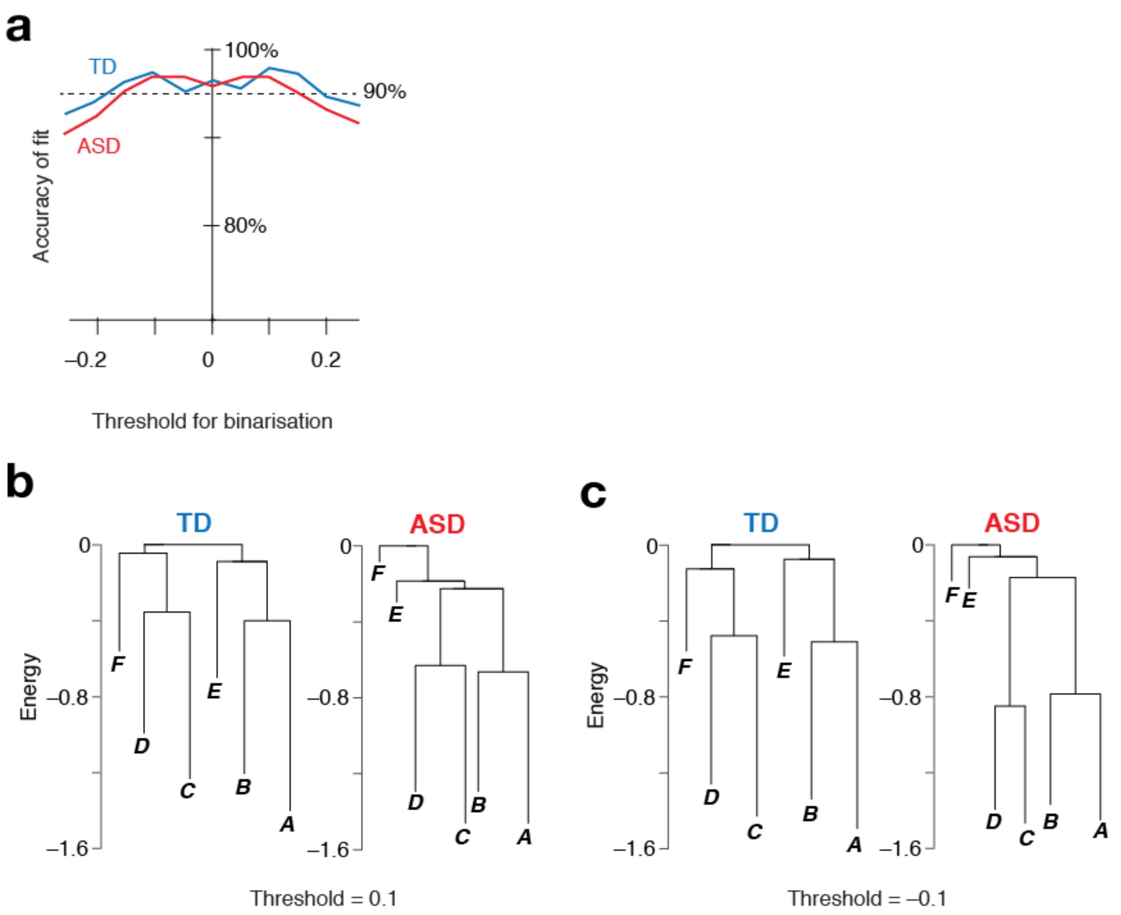

Effects of binarisation thresholds on energy landscape structures.

We examined the robustness of the current observations against changes in the threshold of binarisation of brain activity.

a. As shown in our previous study[1], the accuracy of fit of the pairwise maximum entropy model was higher than 90% as long as the binarisation threshold was in a range of [–0.15, 0.15].

b and **c**. In addition, even when we set the threshold at 1 or –1, the structures of the energy landscapes were qualitatively the same as those obtained by setting the cut-off value at 0. In all the cases, the energy landscapes consisted of the same six brain states (local min A–F), and the minor brain states (local min E and F) had lower energy values in the TD group than in the ASD group.

Supplementary Figure 3

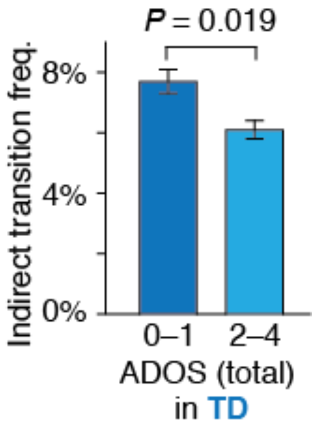

Indirect transition frequency in autistic TD individuals.

As seen in the ASD group, the TD individuals with relatively higher ADOS scores, indicating that they exhibit autistic trait, showed a significantly small indirect transition frequency than those with a lower ADOS scores. This comparison was performed using 16 of the 24 TD individuals, who were given ADOS scores in the ABIDE dataset.

Supplementary Figure 4

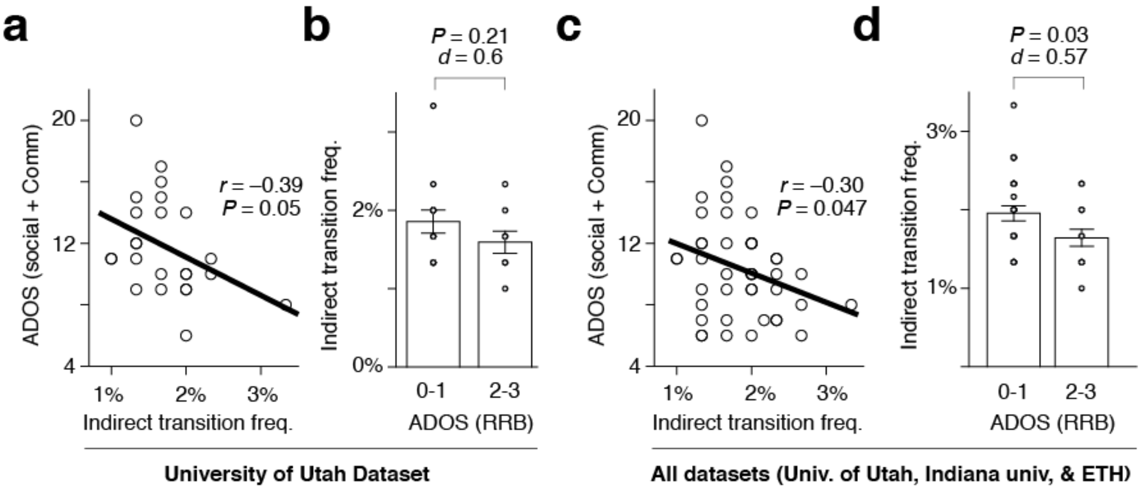

Associations between the indirect transition frequency and sub-scale scores of ADOS.

a. The social ADOS scores showed mild negative associations with the indirect transition frequency in the ASD group.

b. The indirect transition frequency was mildly reduced in the ASD individuals with higher non-social ADOS scores compared to those with lower non-social ADOS scores.

c and **d**. This tendency did not change even after we re-calculated the associations using all the three datasets. According to DSM-5, we quantified the social symptom of autism by merging ADOS social score and ADOS communication score. The correlations between the merged ADOS scores and brain dynamics were preserved when we calculated the correlations for each ADOS subscale separately (ADOS social $r \leq -0.32$, ADOS communication $r \leq -0.41$).

Supplementary Figure 5

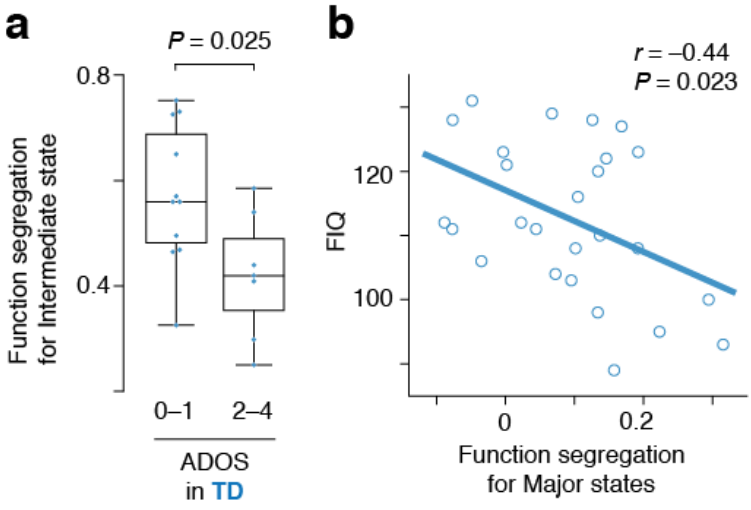

Associations between brain dynamics and behavioural indices in the TD individuals.

a. As seen in the ASD group, the TD individuals with relatively high autistic traits showed significantly low functional segregation during the intermediate state. This analysis was performed using 16 of the 24 TD individuals, who were given ADOS scores in the ABIDE dataset.

b. In the TD individuals, their FIQ was not positively but negatively significantly correlated with the strength of functional segregation for the major states.

Supplementary Figure 6

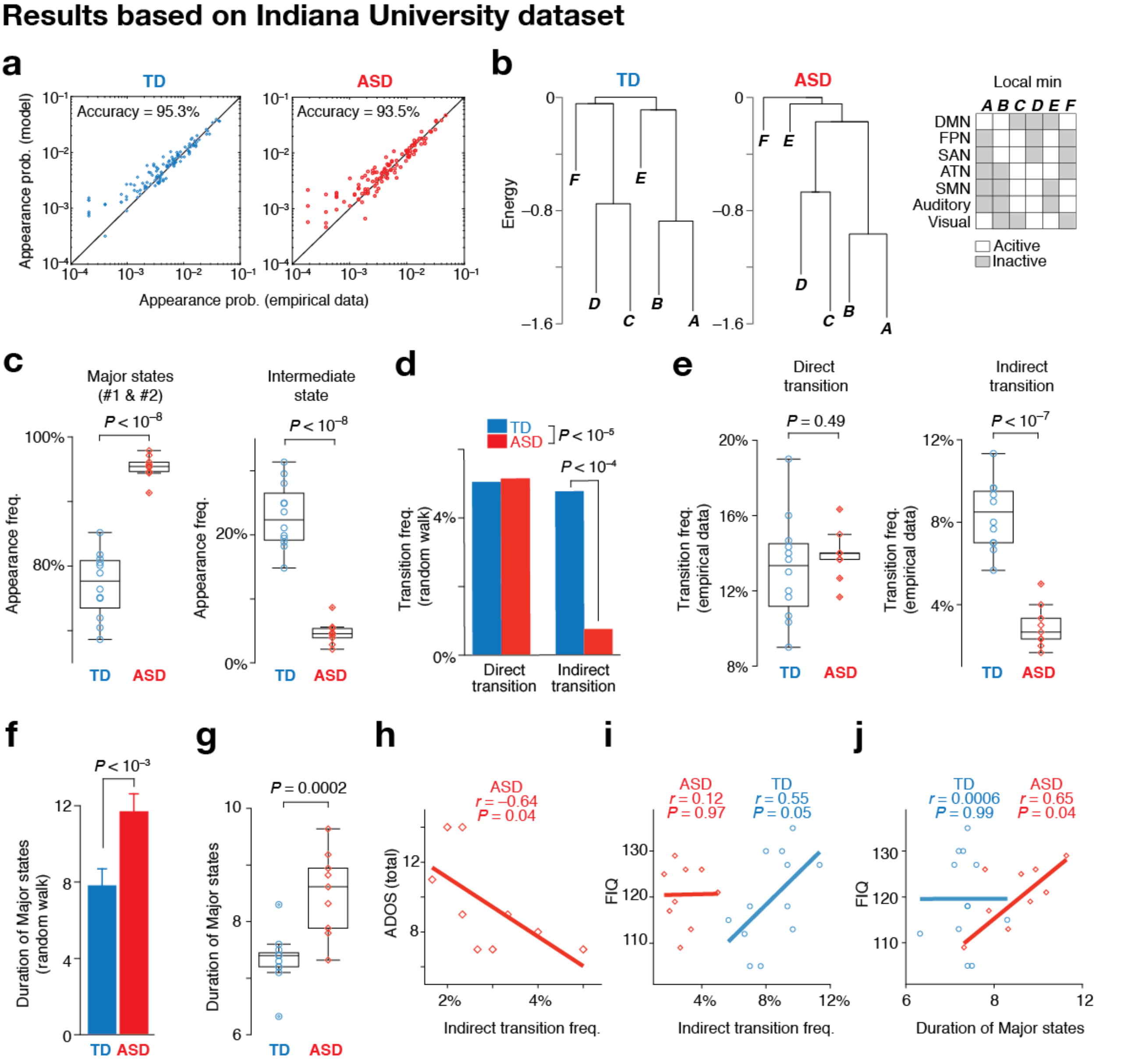

Resting-state brain dynamics seen in the Indiana University Dataset.

Using the dataset collected in the Indiana University (Supplementary Table 2), we observed qualitatively the same results as those based on the original dataset recorded in the University of Utah.

First, the pairwise MEM was accurately fitted to this independent dataset (≥93.5%) (**a**), and yield energy landscapes with qualitatively the same hierarchal structures consisting of the same six local minima (**b**). As in the original results, the appearance frequency of the major states was larger in the ASD group, whereas that of the intermediate state was large in the TD individuals (**c**). In addition, the significant low frequency of the indirect transition and longer duration of the major state in the ASD group were also reproduced (**d-g**). These indices for the bran dynamics were correlated with ADOS total scores and FIQ scores in the same manner as see in the original dataset (**h-j**).

Supplementary Figure 7

Results based on Indiana University dataset

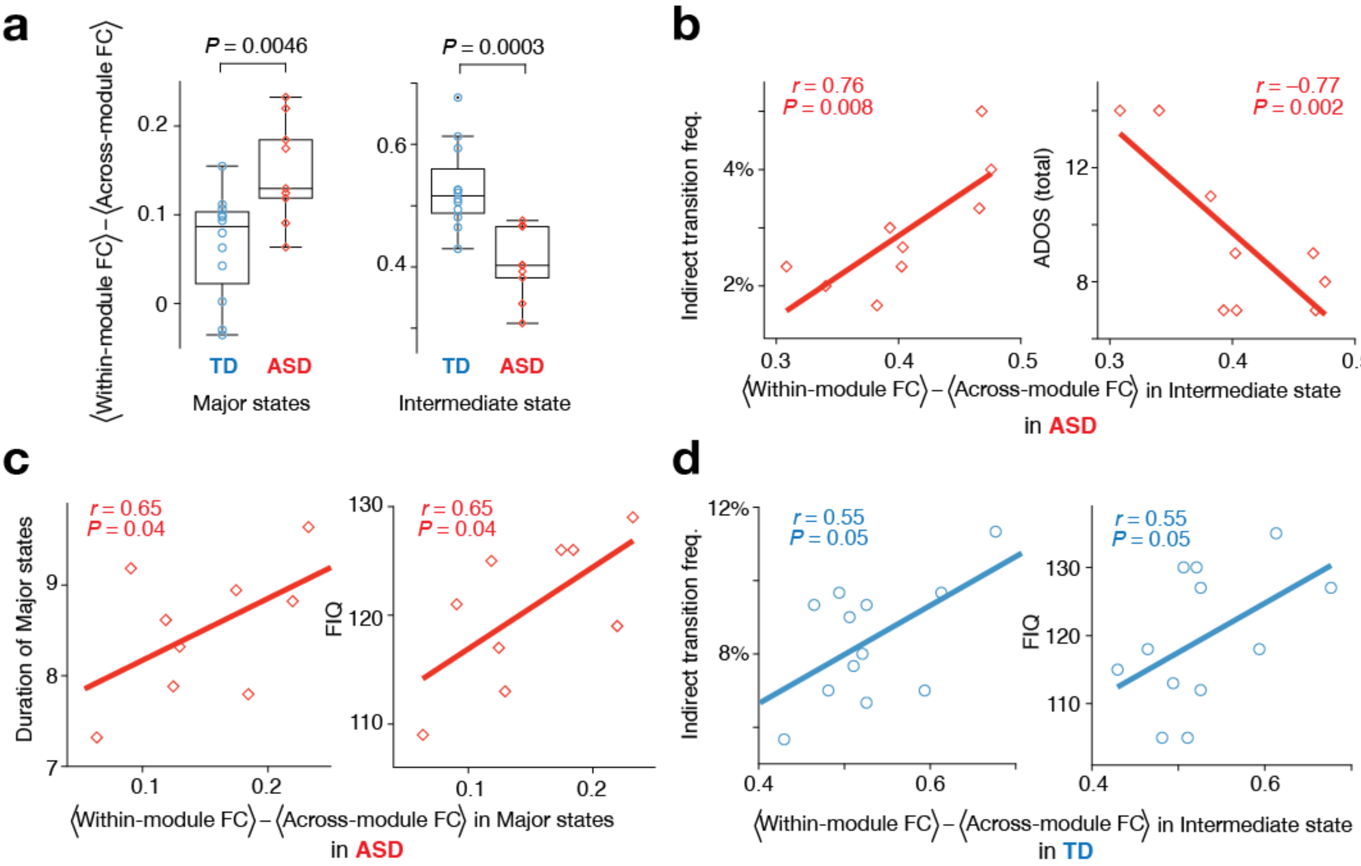

Associations between functional segregations, brain dynamics, and behaviours in the Indiana University dataset.

The results about functional segregations during the major/intermediate states were qualitatively reproduced in the dataset collected in Indiana University dataset.

a. The functional segregation strength for the major states was significantly larger in the ASD data, whereas that for the Intermediate state was larger in the TD group.

b. Such atypically reduced functional segregation for the intermediate state in the ASD individuals were correlated with the ADOS total scores.

c. The atypically elevated functional segregation for the Major states was associated with general cognitive skills in the ASD groups.

d. The general cognitive ability in the TD individuals were correlated the strength of the functional segregation during the intermediate state.

Supplementary Figure 8

Results based on ETH Zürich dataset

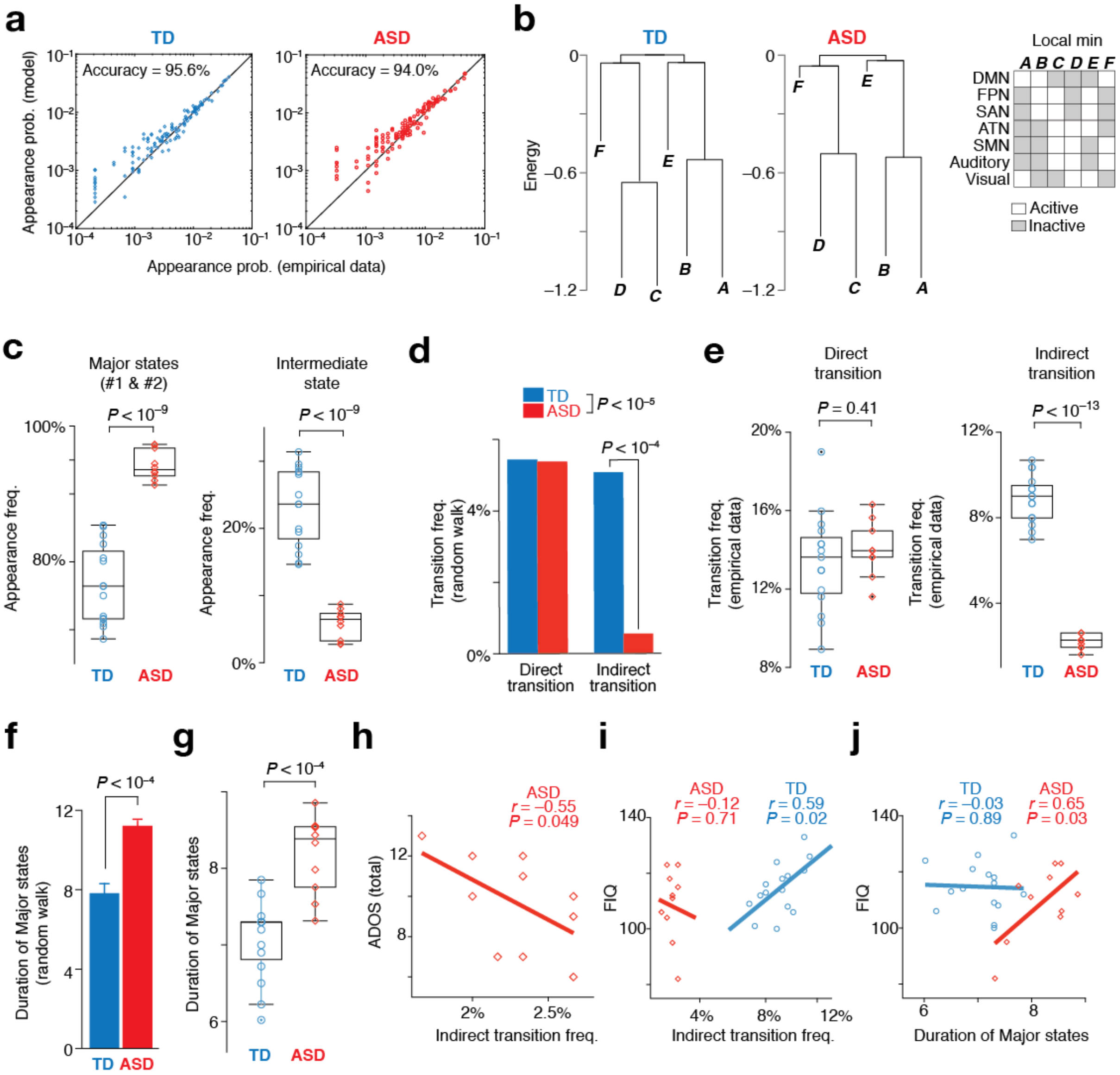

Resting-state brain dynamics seen in the ETH Zürich dataset.

Using the resting-state fMRI dataset collected in the ETH Zürich (Supplementary Table 3), we observed qualitatively the same results as those based on the original dataset recorded in the University of Utah.

First, the pairwise MEM was accurately fitted to this independent dataset (≥94.0%; panel **a**), and yield energy landscapes with qualitatively the same hierarchal structures consisting of the same six local minima (panel **b**). As in the original results, the appearance frequency of the major states was larger in the ASD group, whereas that of the intermediate state was large in the TD individuals (**c**). In addition, the significant low frequency of the Indirect transition and longer duration of the major state in the ASD group were also reproduced (**d-g**). These indices for the bran dynamics were correlated with ADOS total scores and FIQ scores in the same manner as see in the original dataset (**h-j**).

Supplementary Figure 9

Results based on ETH Zürich dataset

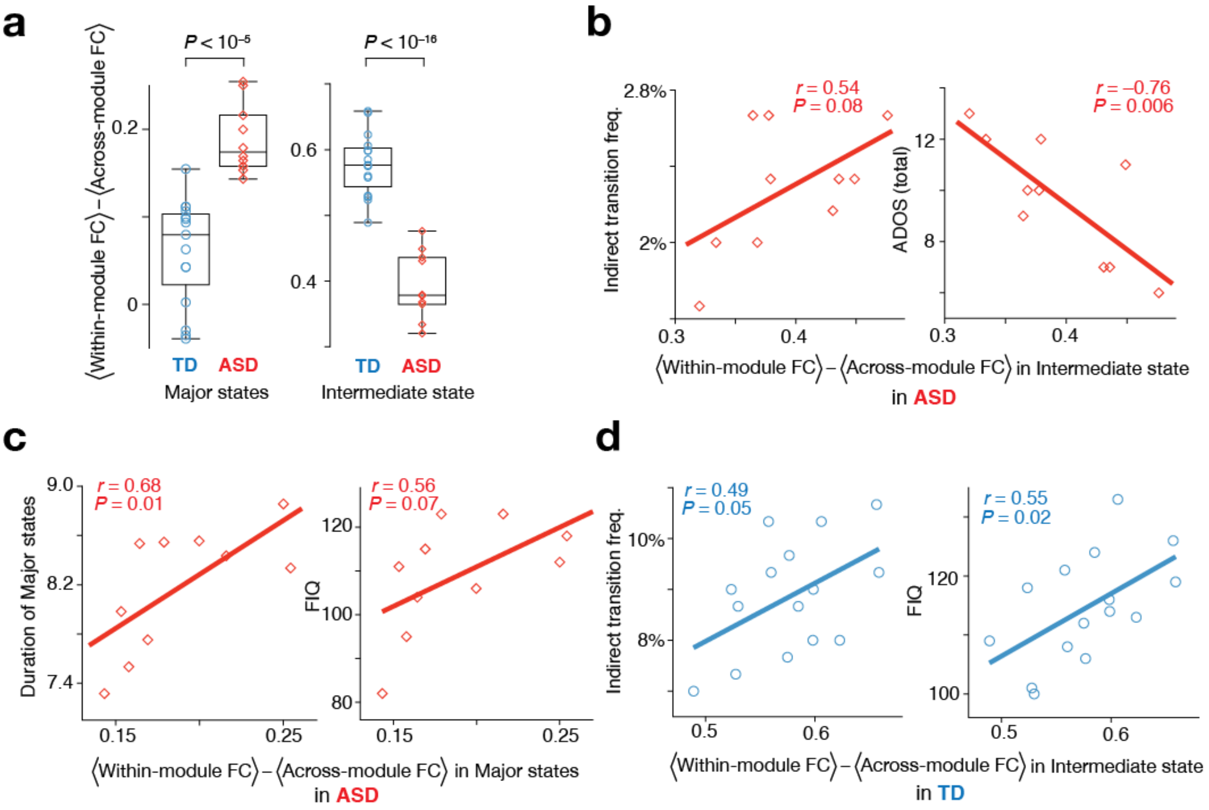

Associations between functional segregations, brain dynamics, and behaviours in the ETH Zürich dataset.

The results about functional segregations during the major/intermediate states were qualitatively reproduced in the dataset collected in ETH Zürich dataset.

a. The functional segregation strength for the major states was significantly larger in the ASD data, whereas that for the intermediate state was larger in the TD group.

b. Such atypically reduced functional segregation for the intermediate state in the ASD individuals were correlated with the ADOS total scores.

c. The atypically elevated functional segregation for the major states was associated with general cognitive skills in the ASD groups.

d. The general cognitive ability in the TD individuals were correlated the strength of the functional segregation during the intermediate state.

Supplementary Figure 10

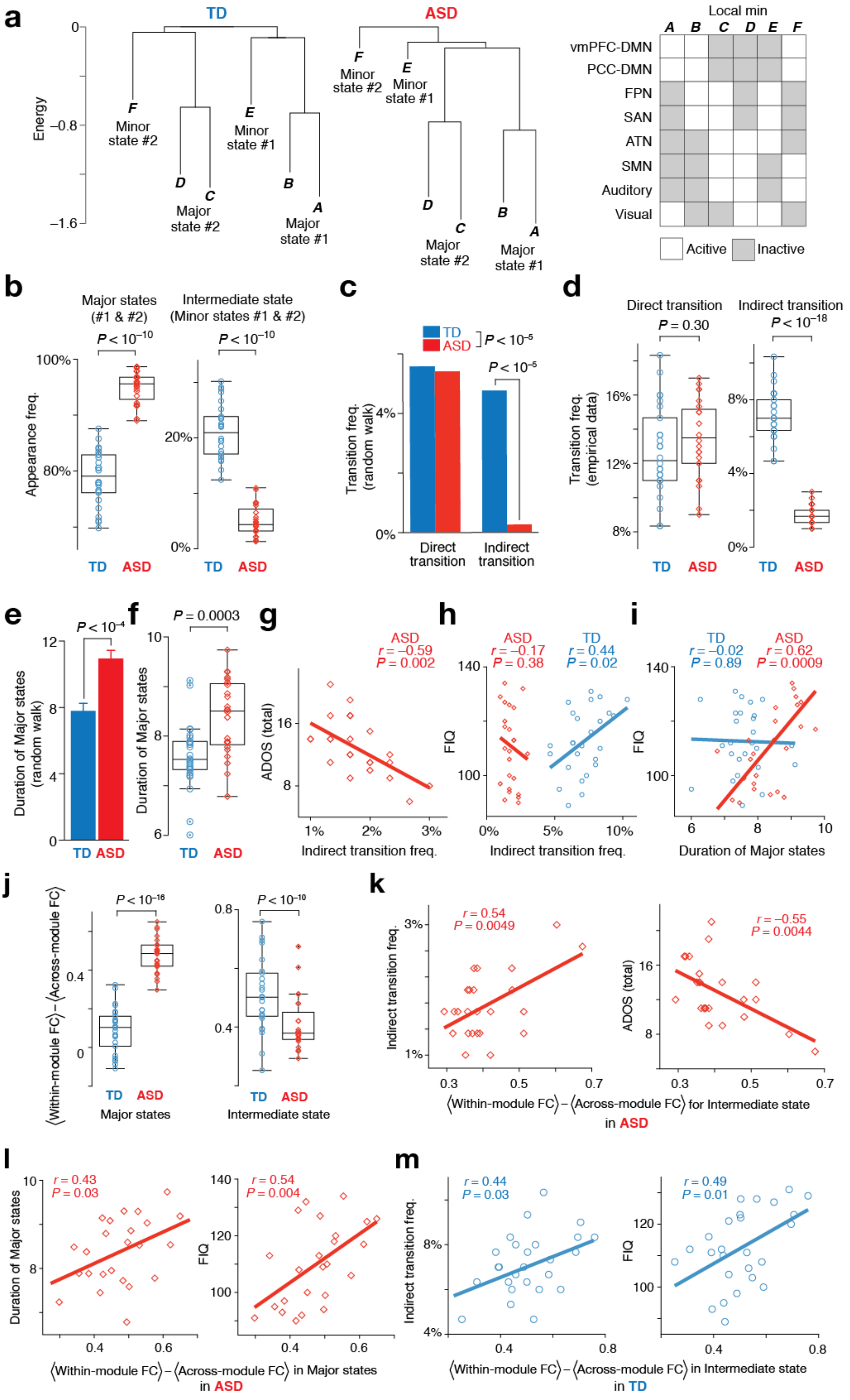

Results based on a finer brain parcellation.

Even after dividing the DMN into two subnetworks (vmPFC-DMN and PCC-DMN)[2], the original observations were qualitatively preserved.

Although the fitting accuracy of the pairwise MEM was slightly reduced due to the increases in the number of the brain networks (82.1% for TD and 80.7% for ASD), we found the newly-obtained energy landscapes have qualitatively the same hierarchal structures with six local minima (**a**). In particular, the two DMN subnetworks showed the same activity patterns in the six local minima, and thus, we could regard the six local minima as qualitatively the same as those seen in the original analysis (Fig. 2b).

As in the original results, the appearance frequency of the major states was larger in the ASD group, whereas that of the intermediate state was large in the TD individuals (**b**). In addition, the significant low frequency of the indirect transition and longer duration of the major state in the ASD group were also reproduced (**c-f**). These indices for the bran dynamics were correlated with ADOS total scores and FIQ scores (**g-i**) in the same manner as see in the original analysis.

In addition, the results about functional segregations during the major/intermediate states (Figs. 6-8) were also reproduced. The functional segregation strength for the major states was significantly larger in the ASD data, whereas that for the intermediate state was larger in the TD group (**j**).

Such atypically reduced functional segregation for the intermediate state in the ASD individuals were correlated with the ADOS total scores (**k**). The atypically elevated functional segregation for the major states was associated with general cognitive skills in the ASD groups (**l**). The general cognitive ability in the TD individuals were correlated the strength of the functional segregation during the intermediate state (**m**).

Supplementary Figure 11

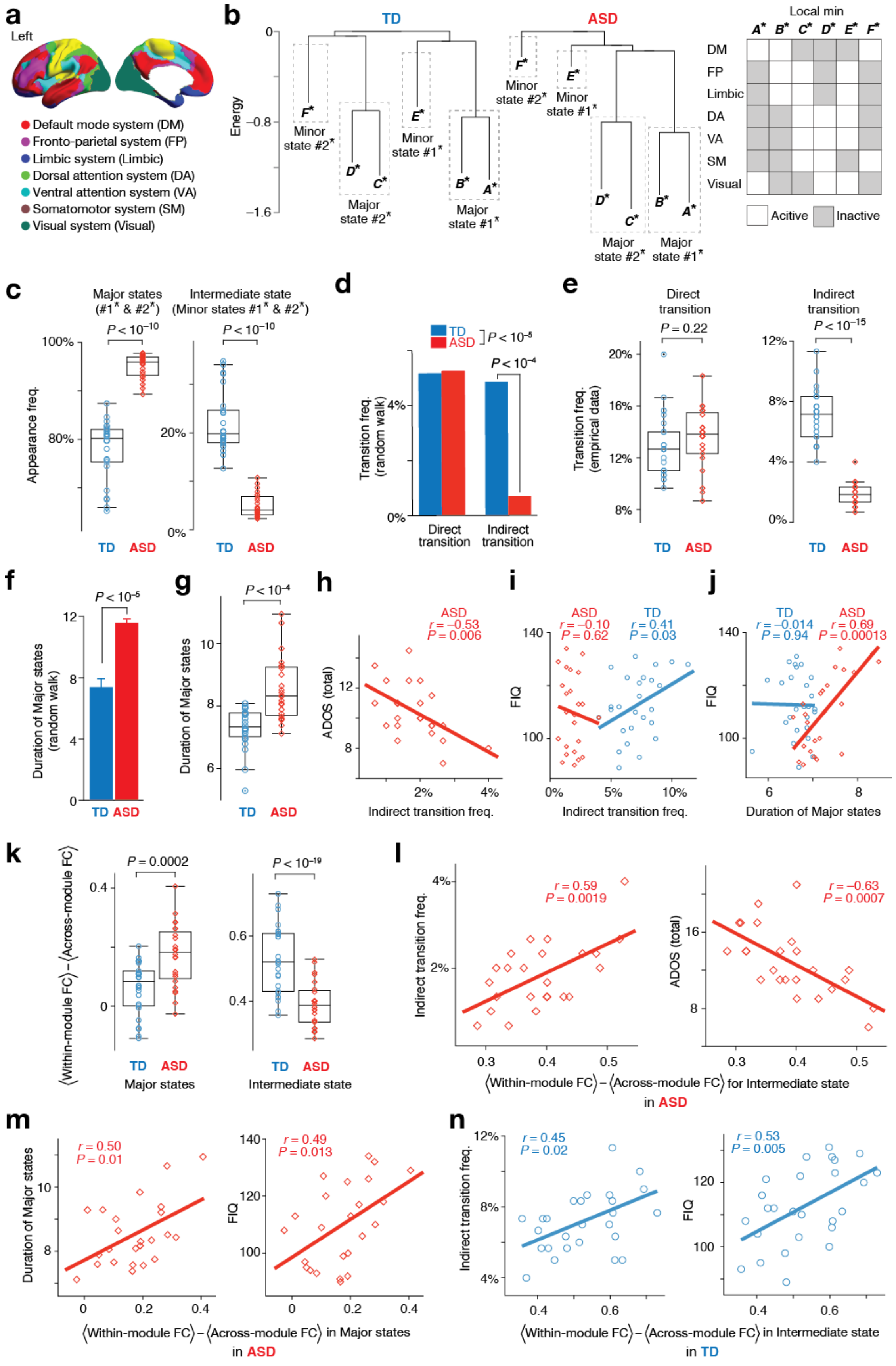

Results based on another different brain parcellation.

We examined whether the original observations were qualitatively reproduced even after the brain cortical area was differently parcellated. In this reproducibility test, we adopted an independent 7-network brain parcellation method proposed by Yeo et al[3] (DM, FP, Limbic, DA, VA, SM and Visual) (**a**).

First, we found that the pairwise MEM was accurately fitted (95.5% for TD and 94.2% for ASD), and yielded energy landscapes with the seemingly same hierarchal structures consisting of six local minima (**b**).

Given that Limbic system anatomically overlaps with the SAN in the original brain parcellation and SM system almost includes the original Auditory network, the six local minima *A**-*F** found here can be considered to correspond to the original six local minima A-F, respectively. If this approximation is allowed, the energy landscapes can be seen as qualitatively the same as those obtained in the original analyses.

Moreover, the other original findings were also qualitatively reproduced in this different brain parcellation.

The appearance frequency of the major states was larger in the ASD group, whereas that of the intermediate state was large in the TD individuals (**c**). In addition, the significant low frequency of the indirect transition and longer duration of the major state in the ASD group were also reproduced (**d-g**). These indices for the bran dynamics were correlated with ADOS total scores and FIQ scores (**h-j**) in the same manner as see in the original analysis.

In addition, the results about functional segregations during the major/intermediate states were also reproduced. The functional segregation strength for the major states was significantly larger in the ASD data, whereas that for the intermediate state was larger in the TD group (**k**).

Such atypically reduced functional segregation for the intermediate state in the ASD individuals were correlated with the ADOS total scores (**l**). The atypically elevated functional segregation for the major states was associated with general cognitive skills in the ASD groups (**m**). The general cognitive ability in the TD individuals were correlated the strength of the functional segregation during the intermediate state (**n**).

Supplementary Figure 12

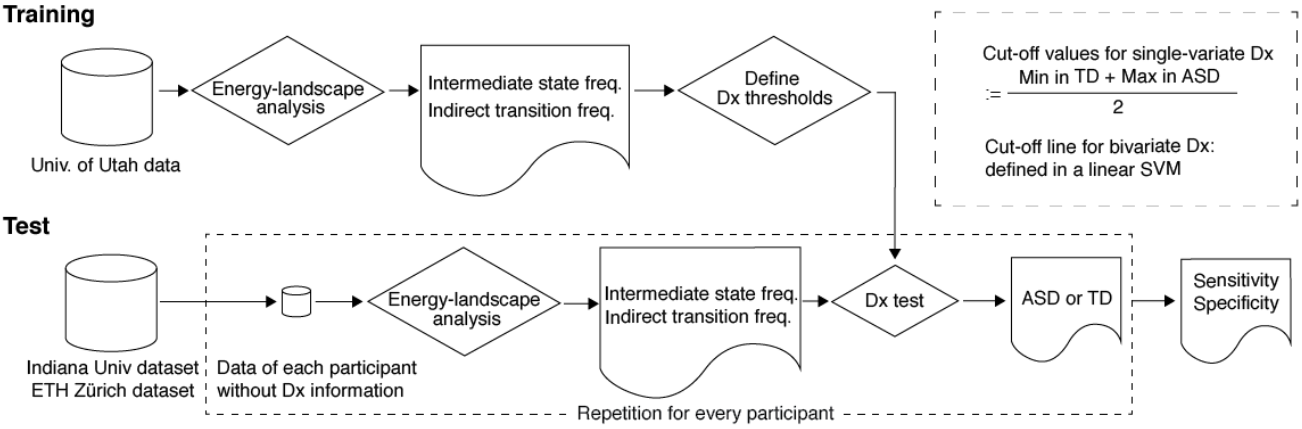

Procedure for diagnosis prediction based on brain dynamics.

The diagnosis prediction was performed in two manners: single-variate and bi-variate methods. In both methods, the diagnosis thresholds were defined using the University Utah dataset, and their classification accuracies were evaluated with independent datasets collected in the Indiana University (Supplementary Table 2) and ETH Zürich (Supplementary Table 3).

The intermediate state frequency and indirect transition frequency were adopted for this diagnosis prediction, because they were substantially different between the TD and ASD groups.

Supplementary Figure 13

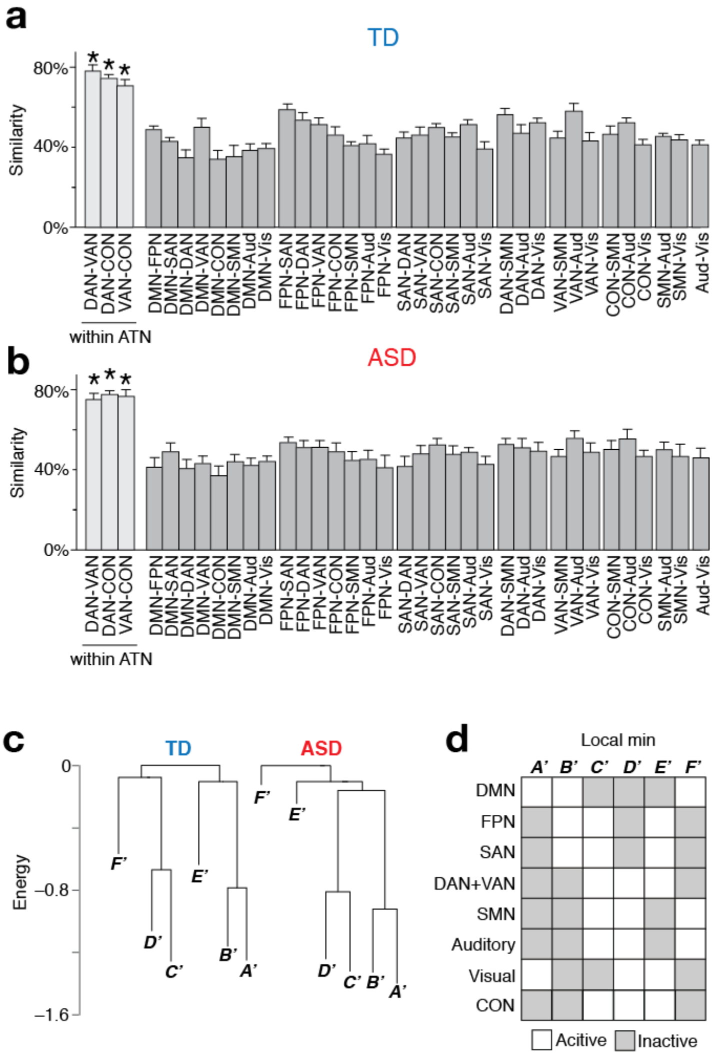

Justification for merging DAN, VAN, and CON into one brain system.

a and **b.** We examined the similarity in the brain activity between the three brain systems (dorsal/ventral attention networks and cingulo-opecular network) that constitute the Attention Network (ATN). Technically, we compared binarised network activity between the nine cortical brain networks because the current energy-landscape analysis was performed based on such binary data. The similarity between the binary network activities was calculated for every pair of the nine brain networks for each participant.

We found that in both TD group (**a**) and ASD group (**b**), the similarity was selectively higher within the three networks (DAN, VAN, and CON) than the other pairs of the networks. The similarity within the three networks was statistically higher than a chance level (≥71% in TD, ≥73% in ASD, $P \leq 10^{-5}$ in one-sample t-tests across participants). The other pairs of the brain networks were not significantly high (<58% in TD and ASD, $P > 0.05$ in one-sample t-tests). Error bars indicate standard deviation. *, $P < 0.05$.

c and **d**. We examined energy landscapes after dividing the ATN into the CON and the other two systems (i.e., DAN and VAN). Although the accuracy of fit was decreased (~83%), qualitatively the same energy landscapes with the same six local minima were observed as in the original analysis. Note that in the six local minima (**d**), the CON showed the same activity patterns as the DAN+VAN did.

Supplementary Figure 14

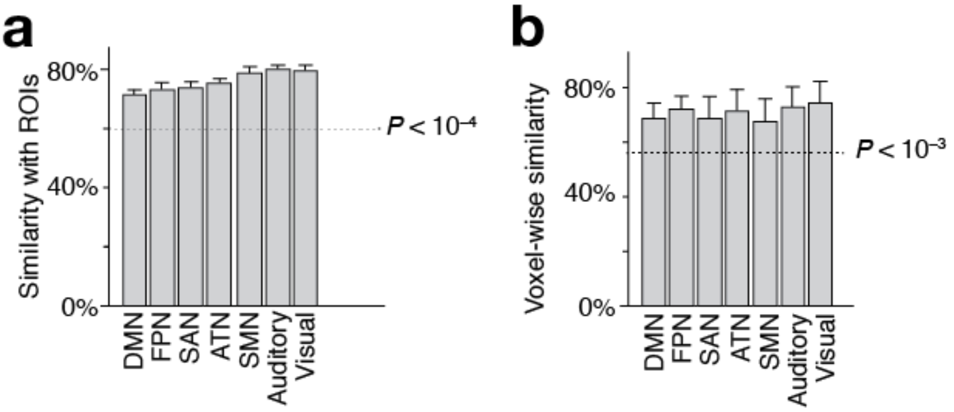

Similarity between network activity and ROI-based/voxel-based activity.

The current energy-landscape analysis was based on the average brain network activity across ROIs in each network.

In a binary form, the network activity was significantly similar to ROI-based brain activity (≥69%, $P < 10^{-4}$ in a binominal test) (**a**). In addition, the network activity was sufficiently similar to voxel-wise brain activity (≥66%, $P < 10^{-3}$ in a binominal test) (**b**).

Supplementary Figure 15

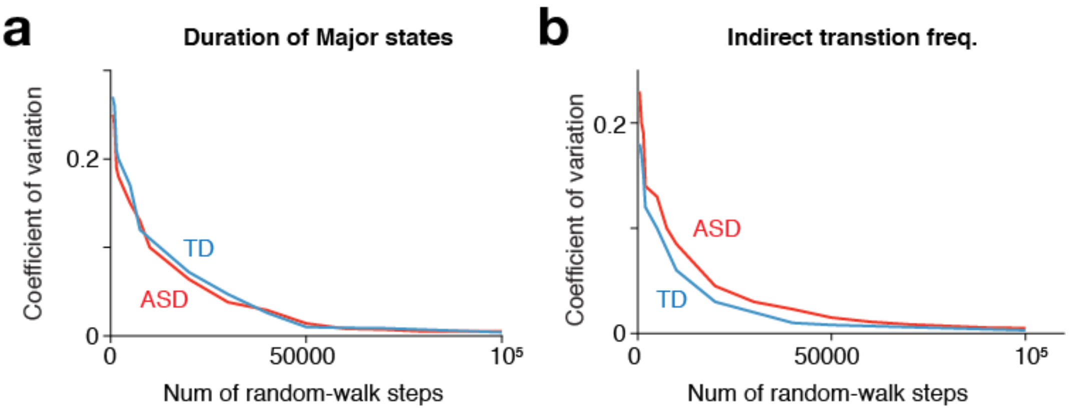

Associations between the number of random-walk steps and the coefficients of variation of the tow brain dynamics indices.

In the two brain dynamics indices (duration of major states and indirect transition frequency), as the number of the random-walk steps increased, the coefficients of variation (:= standard deviation/mean) of the indices decreased and reached plateau after approximately 60000 steps (<0.005). Therefore, we set the number of the steps in the current random-walk simulation at 10^5.

We calculated the coefficients of variation by repeating the random-walk simulation with each number of step 1000 times. The lines in this figure show the average across the 1000 trials.

Supplementary Table 1

Correlation coefficients (*r*) between brain dynamics and age

	Intermediate state freq.	Indirect transition freq.	Duration of Major states
TD	0.18	0.17	–0.12
ASD	–0.13	–0.16	–0.11

Supplementary Table 2

Demographic data of Indiana University dataset

	Typically developing (TD)	Autism spectrum disorder (ASD)	*P* value
Number of participants	12	9	–
Age	23.9±5.8 (19–37) yo	21.5±5.4 (19–37) yo	0.51
Sex	Male	Male	–
Handedness	Right	Right	–
Full IQ	119.6±10.0 (105–135)	120.6±6.7 (109–129)	0.8
Verbal IQ	115.9±10.4 (105–139)	123.3±14.6 (98–138)	0.21
Performance IQ	118.9±12.7 (99–140)	111.7±7.8 (102–127)	0.2
Head motion	1.2±0.4 (0.31–2.8) mm	1.4±0.5 (0.25–2.4) mm	0.41
ADOS total	–	9.6±2.8 (7–14)	–
ADOS Social	–	5.7±1.2 (4–8)	–
ADOS Communication	–	2.2±1.3 (1–5)	–
ADOS RRB	–	1.7±1.0 (0–3)	–

Mean ± SD (Min–Max)

Supplementary Table 3

Demographic data of ETH Zürich dataset

	Typically developing (TD)	Autism spectrum disorder (ASD)	*P* value
Number of participants	15	10	–
Age	23.2±3.3 (18–29) yo	21.5±2.7 (18.5–27.25) yo	0.2
Sex	Male	Male	–
Handedness	Right	Right	–
Full IQ	114.7±9.2 (100–133)	108.9±12.8 (82–123)	0.21
Verbal IQ	115±12.1 (97–139)	109.5±14.5 (85–129)	0.23
Performance IQ	112.7±10.1 (96–133)	107.0±11.9 (84–123)	0.21
Head motion	1.2±0.8 (0.13–2.3) mm	1.5±0.9 (0.15–2.4) mm	0.62
ADOS total	–	9.7±2.4 (6–13)	–
ADOS Social	–	6.7±1.3 (5–9)	–
ADOS Communication	–	2.5±1.1 (1–5)	–
ADOS RRB	–	0.5±0.97 (0–3)	–

Mean ± SD (Min–Max)

Supplementary Table 4

Correlation coefficients (*r*) between functional segregation strength and global FC level.

	Sum of |FC|	Sum of FCs	Sum of positive FCs	Sum of negative FCs
TD				
Functional segregation in Major states	0.082	–0.16	–0.021	–0.16
Functional segregation in Intermediate state	–0.075	0.040	–0.039	0.088
ASD				
Functional segregation in Major states	0.13	–0.0066	0.15	–0.11
Functional segregation in Intermediate state	0.073	–0.040	0.06	–0.072

Sum of |FC|: summation of the absolute values of FCs.
Sum of FCs: summation of FCs.
Sum of positive FCs: summation of only positive FCs.
Sum of negative FCs: summation of only negative FCs.

These summations were performed using Fisher-transformed FCs (i.e., *Z* value).

Supplementary references

1. Watanabe, T. *et al.* A pairwise maximum entropy model accurately describes resting-state human brain networks. *Nat Commun* **4,** 1370 (2013).
2. Uddin, L. Q., Kelly, A. M., Biswal, B. B., Castellanos, F. X. & Milham, M. P. Functional connectivity of default mode network components: correlation, anticorrelation, and causality. *Hum Brain Mapp* **30,** 625–637 (2009).
3. Yeo, B. T. T. *et al.* The organization of the human cerebral cortex estimated by intrinsic functional connectivity. *Journal of Neurophysiology* **106,** 1125–1165 (2011).
